# Angioleiomyoma originating from the ano-rectal wall presenting as a perineal mass: a case report

**DOI:** 10.1007/s00384-025-04836-7

**Published:** 2025-02-24

**Authors:** Sayali Valiyeva, Elena Cicerone, Elisabetta Iacobelli, Gina R. Quaglione, Renato Pietroletti

**Affiliations:** 1 Department of Applied Clinical & Biotechnological Sciences, Unit of Surgical Coloproctology Hospital Val Vibrata, Sant’Omero (TE), University of L’Aquila, L’Aquila, 67100 Italy; 2 Unit of Pathology Hospital “Mazzini” Teramo, Teramo, Italy

**Keywords:** Angioleiomyoma, Rectum, Vascular leiomyoma, Case report

## Abstract

**Introduction:**

Angioleiomyoma, a vascular leiomyoma is a rare, benign smooth-muscle tumor observed to occur anywhere in the body, most frequently in the lower extremities but very rarely in the digestive system. Angioleiomyoma in the hindgut is infrequent and in particular, rectal/perianal location has been observed very rarely.

**Case report:**

We describe herein a case of a 50-year-old male patient complaining of perineal discomfort and a swelling at the level of the left ischio-rectal fossa, moderately painful. This solid mass in the left ischio-rectal space was in close relationship with the wall of the ano-rectal junction. After surgical removal and histopathology, the mass resulted an angioleiomyoma, vascular type, desmin positive, a very rare neoplasm. Extensive immune-histochemical studies are fundamental for the correct diagnosis and to rule out other mesenchymal tumors.

Discussion/conclusion.

Angioleiomyoma is a very rare neoplasm of the gastrointestinal tract, and the fundamental problem of peri-rectal/perianal angioleiomyoma is represented by differential diagnosis from gastrointestinal stromal tumors (GISTs) and other perianal/perirectal swellings. For correct differential diagnosis, the histopathology supported by extensive immune-histochemical study adopting a panel of specific tissue markers is important. The surgical treatment is mandatory with complete excision and subsequent follow-up since local recurrence may occur.

**Supplementary Information:**

The online version contains supplementary material available at 10.1007/s00384-025-04836-7.

## Introduction

Angioleiomyoma, a vascular leiomyoma, is a rare mesenchymal neoplasia that originates in the tunica media of vessels [1]. This type of vascular neoplasia should be considered a variety of deep soft tissue leiomyoma according to Kilpatrick classification [2]. This benign smooth-muscle tumor has been observed in a wide age range, with a peak in the 4th to 6th decades of life with women more commonly affected than men [3–6]. Angioleiomyoma has been reported to occur anywhere in the body, frequently in the lower extremities [7, 8]. The location in the digestive tract has been observed mainly in the stomach and in the small bowel, whereas the large bowel and rectum are rarely involved [9]. We report herein a case of mesenchymal neoplasia detected in the left ischio-rectal space and closely adherent to the wall of the ano-rectal junction. After surgical removal, the neoplasm was diagnosed as angioleiomyoma, also with the help of an extensive panel of immunocytochemical staining. Perianal/perirectal location of an angioleiomyoma [10, 11] is considered quite exceptional since it is reported in less than 0.1% of the cases or in 1:2000 rectal neoplasms [12]. We report herein a rare case of perineal mass resulting as an angioleiomyoma, a very rare mesenchymal tumor.

## Case report

A 50-year-old man came to our attention complaining of perineal discomfort and a swelling at the level of the left ischio-rectal fossa, moderately painful. The patient’s difficulties began 12 months earlier, in the form of a small lump appearing in the left perianal region with a sense of discomfort in the anus. During the last 3 months, the lesion grew up and became moderately painful especially when sitting on hard surfaces. For these reasons, he decided to undergo a proctological examination. The patient appeared in good health, with no medical history. General physical examination was unremarkable and blood tests were normal, and a colonoscopy performed 1 year before in a screening program resulted negative.

The inspection of the perineum resulted unremarkable. On palpation, a solid mass was felt in the left ischio-rectal region, tender, mobile, and elastic. On digital rectal examination, the swelling could be appreciated starting from the dentate line, extending 2–3 cm upwards in very close relationship with the wall of the ano-rectal junction, occupying about 1/3 of the circumference. The wall of the anus and the rectum did not seem infiltrated but only compressed. Anoscopy and recto-sigmoidoscopy resulted normal.

**Fig. 1:**
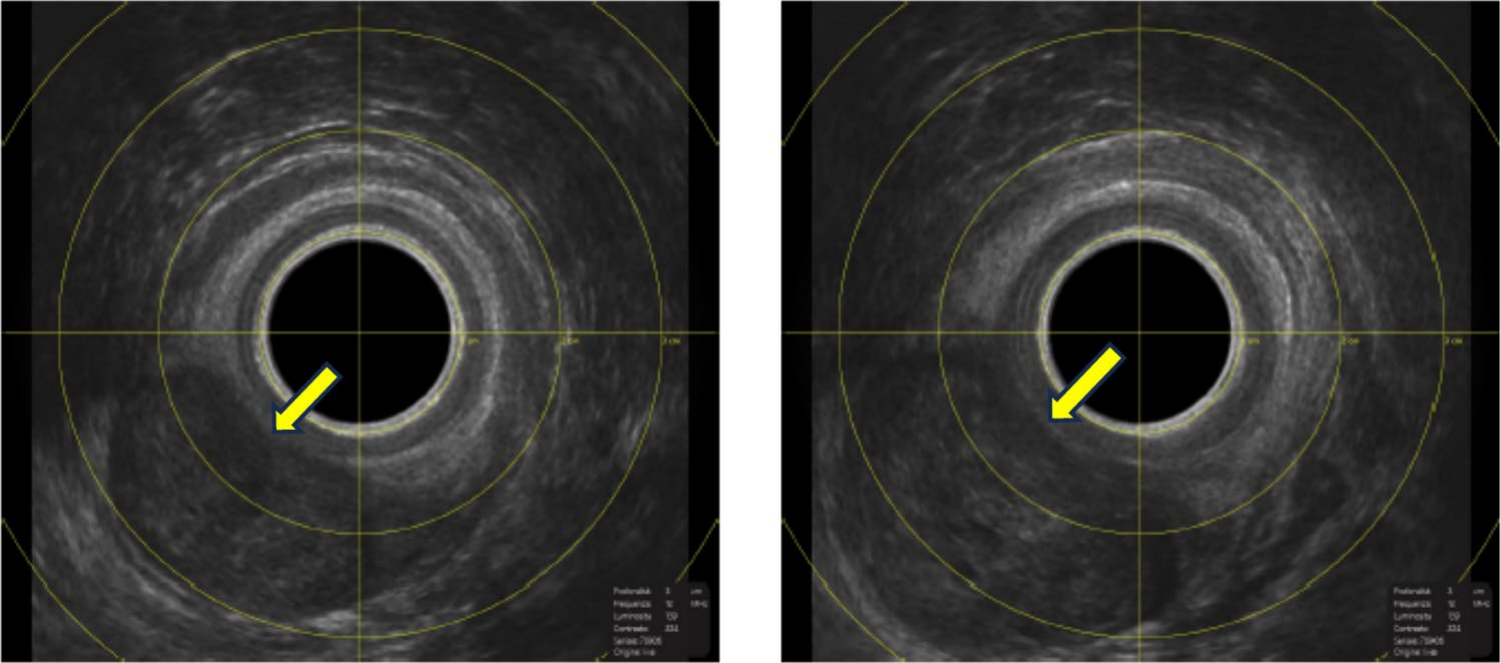
EA-US in left-lateral position. A hypoechoic, dis-homogeneous mass in the left ischio-rectal space (yellow arrows). The external sphincter is interrupted from three to six o’clock

At endoanal ultrasound (EA-US), in the middle/upper part anal canal, a hypoechoic and pluri-lobulated mass measuring 4.9 × 3.5 cm was detected in the left ischio-rectal space, extending from three to six o’clock in lithotomy. The mass showed a dis-homogeneous thickening very close to the anal canal. At this level, the lesion seemed to originate from the external anal sphincter (Fig. 1).

Colorectal cancer markers resulted negative. Based upon clinical (soft, smooth mass, mobile, well defined) and ultrasound features, we opted for immediate surgical excision to obtain histopathology and diagnosis.

**Fig. 2:**
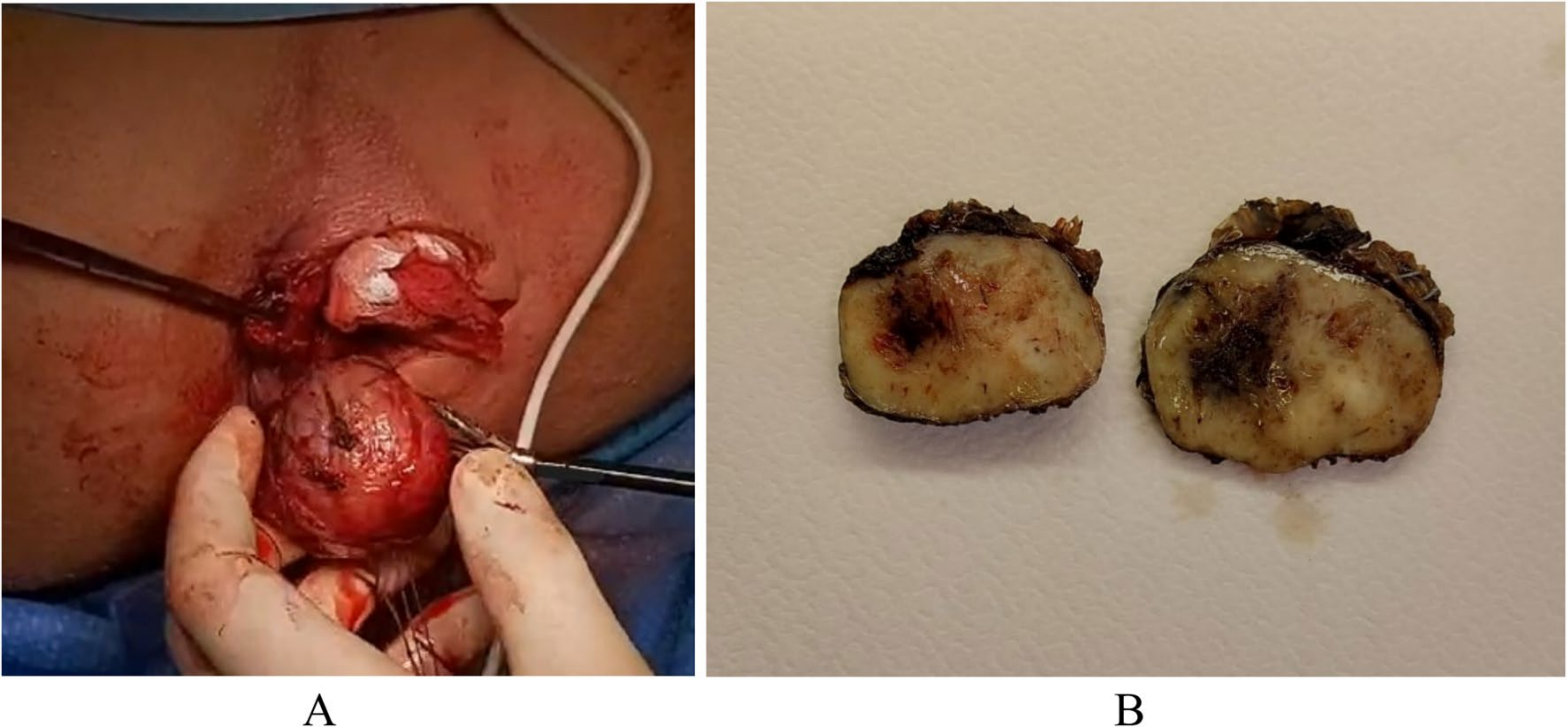
**A** Intraoperative image of round, well capsulated formation. **B** Macroscopic appearance of the specimen, with central hemorrhagic area

The operation was carried out in lithotomy position under spinal anesthesia. Bowel preparation was performed by means of enemas administered the evening before the operation and early morning in the day of the operation. “One-shot” antibiotic prophylaxis was administered intravenously with 500 mg of metronidazole. After skin disinfection with povidone iodine solution and draping, a transverse incision on the perianal skin was made in the left ischio-rectal fossa. The ischio-rectal space was entered, the capsulated mass was detected and severed out from the fat of the ischio-rectal fossa by means of gentle traction and sharp dissection. The rounded, capsulated mass was in close proximity to the wall of the ano-rectal junction and adherent on the left side of the upper external sphincter, where the vascular pedicle of the mass seemed to originate (Fig. 2A). At this level, the dissection was performed with the aid of a radiofrequency device (Caiman B-Braun®). Complete removal was achieved without damage to the surrounding structures, in particular the rectal wall and the external sphincter. The wound was closed in double layer with interrupted sutures, leaving a Penrose drain removed after 24 h.

**Fig. 3:**
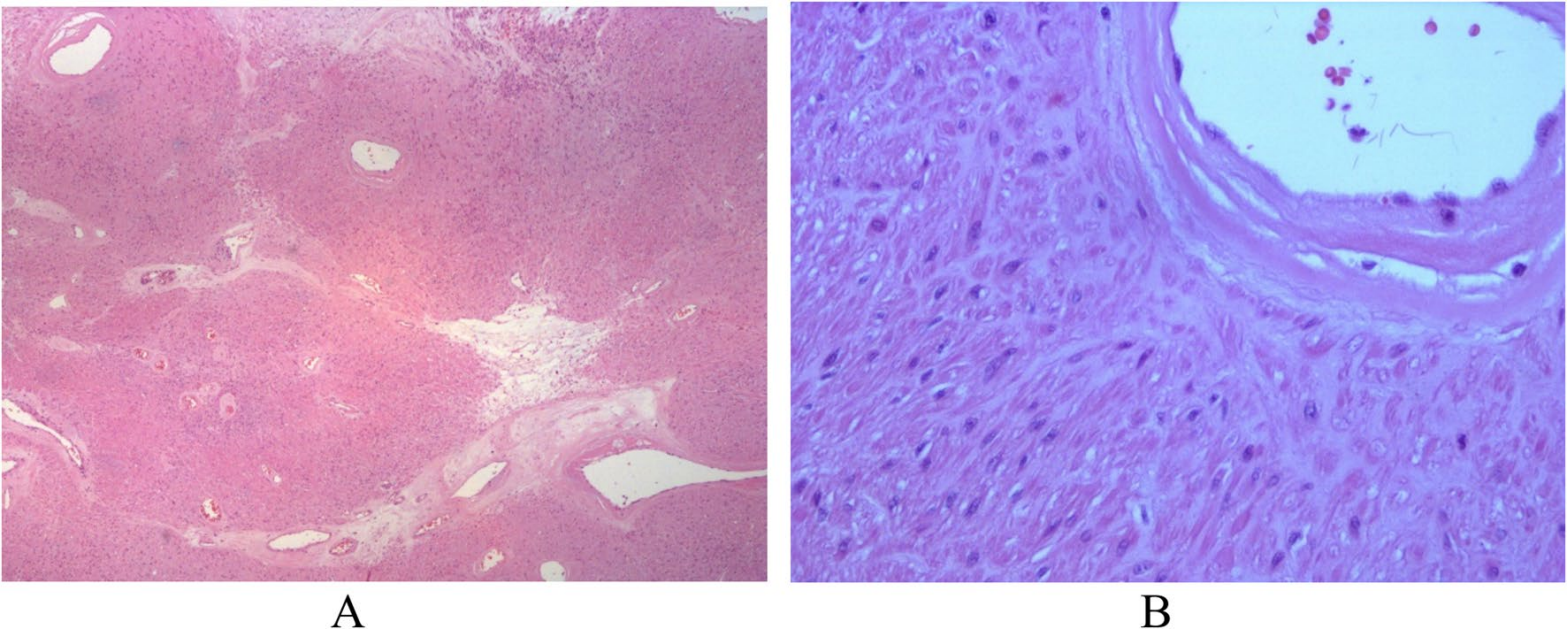
H&E staining of 3µ sections. Nests of spindle shaped cells surrounding vascular structures. No evidence of necrosis, mitotic activity, or atypia (**A** × 5, **B** × 40)

Macroscopic examination found a solid, sharply circumscribed neoplasm, with a white-yellowish cut surface and a central hemorrhagic area (Fig. 2B). The tissue sample was formalin fixed and paraffin embedded. Histological examination at H&E staining detected thick muscle-coated blood vessels with bland, well-differentiated smooth muscle cells of the vascular walls, swirling and blending with smooth intervascular bundles. No mitoses neither atypia was observed (Fig. 3A, B). The pathology report diagnosed an angioleiomyoma, venous-type.

**Fig. 4:**
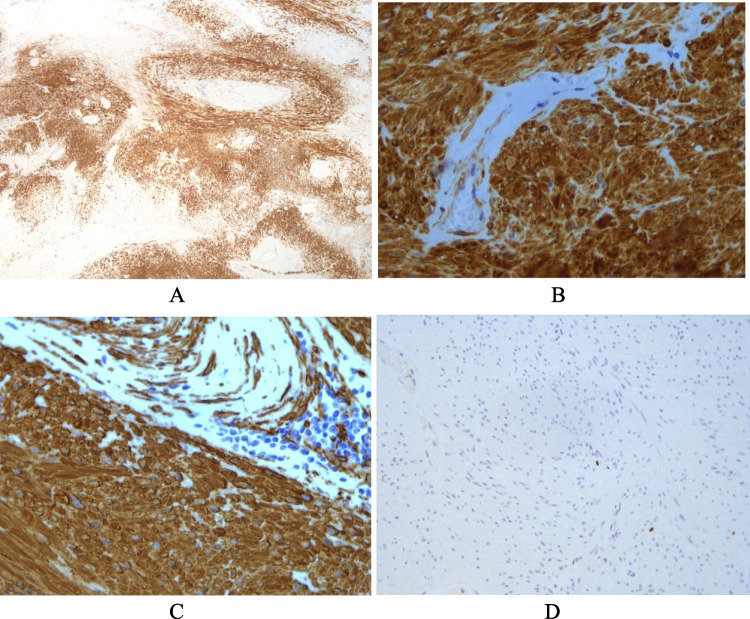
**A** Strong and diffuse positivity for desmin (× 5). **B** Calponin (× 40). **C** Desmin (× 40). **D** Low proliferative activity, ki67 < 1% (× 20)

Immunohistochemical staining for desmin (FLEX monoclonal Mouse anti-Human Desmin ready to use, Clone D33), smooth muscle actin (SMA) (FLEX monoclonal Mouse anti-Human Smooth Muscle Actin, ready to use, Clone 1A4), calponin (FLEX monoclonal Mouse anti-Human Calponin ready to use, Clone CALP), and Ki67 (FLEX monoclonal Mouse anti-Human Ki67 ready to use, Clone MIB1) was performed on DAKO Omnis. The tumor cells resulted diffusely positive for desmin, SMA, and calponin (Fig. 4); ki67/mib1 was < 1%. The marker for gastrointestinal stromal tumors CD117, CD 34, and DOG-1 resulted negative.

The postoperative course was uneventful and the patient was discharged on postoperative day 2. At follow-up visit, 1 month after the operation, the patient was feeling well; surgical scar and digital rectal examination were unremarkable. The patient attended regular follow-up visits at 6 and 12 months from the operation, apparently free from recurrence with normal ano-rectal function.

## Discussion

Angioleiomyoma, also known as vascular leiomyoma, is a rare, benign, solitary neoplasm that originates from the vascular smooth muscle. Angioleiomyomas are very rare in the gastrointestinal tract; they tend to occur mostly in the jejunum (44%) followed by ileum (37%) and duodenum (19%) [13]. They are much less frequent in the esophagus and colon. Leiomyoma in the colon and rectum accounts for 3% of all gastrointestinal benign tumors of smooth muscles [14, 15]. In the literature, there are several publications describing cases with complications, such as bleeding, volvulus, peritonitis, and perforations [13, 16, 17]. Local recurrence has been rarely observed [18]. This neoplasm is rarely diagnosed until histopathological examination is performed after surgical removal.

They originate from the muscle coat or from the sphincter, leaving intact the internal surface of the anal canal or the rectum [19]. Of course, the differential diagnosis in the case of a deep, soft tissue mass can be posed with a variety of benign and malignant stromal tumors such as lipomas, fibromas, myomas, and endometriomas or sarcomas. Recent views tend to consider all mesenchymal tumors as gastrointestinal stromal tumors (GISTs) [19] leaving the final diagnosis to immune-histochemistry. Thus, GISTs are characterized by intense immunostaining for CD117, CD34, and actin and negative for desmin. Conversely, leiomyoma accounts for desmin immunostaining and negative immunoreactivity for CD117 and CD34. Table 1 summarizes the main clinical and immunohistochemical features of the case reported in the literature [12, 19–25], including the present case. This pattern of immune-staining has been observed in our case. If a GIST is identified in a mesenchymal tumor of the anus (less than 2% of the cases), the malignancy potential is high (10–30%) [20]; therefore, follow-up should be adequate.

**Table 1:** Summary of the main clinical and Immunohistochemical features of the case reported in the literature (including present case)

Study	Publication year	Age (yr)/sex	Symptom	Physical examination	Capsule	Immunohistochemistry
Huilgol et al. [20]	2003	42	Painful right buttock mass	A rubbery 9 × 8 × 6-cm mass	Pseudocapsule	Actin and desmin positive CD34 and CD117 negative
Dasari et al. [19]	2007	45/F	Painless swelling	65-mm soft tissue mass	Pseudocapsule	Actin and desmin positive Estrogen and progesterone receptors positive CD117 negative
Kim et al. [21]	2009	30/F	Palpable mass	46 × 35-mm firm mass	Thin pseudocapsule	Actin and desmin positive CD117 and S100 negative
Canda et al. [22]	2010	37/F	Gradually enlarging anal mass	80 × 40-mm solid mass	Capsule	Actin, desmin, and caldesmon positive CD117, CD34, and S100 negative
Alonso Gómez et al. [23]	2011	54/M	Palpable mass	43 × 31-mm solid mass	Fibrous pseudocapsule	Actin and desmin positive CD117, CD34, and S100 negative
Sun et al. [24]	2017	28/F	Perianal mass	70 × 40-mm firm mass	Capsule	Actin, desmin, and calponin positive CD117, CD31, CD34, CD68, CD163, S100, EMA, and Dog1 negative
Dagmura et al. [25]	2019	51/M	History of perianal purulent discharge	35 × 24-mm rubbery mass and external orifice of the perianal fistula	Pseudocapsule	Actin and desmin positive CD117, CD34, and S100 negative
González-Díaz et al. [12]	2021	75/F	Pelvic organ prolapse and urinary incontinence	33 × 27-mm solid mass	Thin pseudocapsule	Actin and desmin positive Estrogen and progesterone receptors positive CD117 and S100 negative
Present case	2023	50/M	Perianal discomfort and swelling	4.9 × 3.5 solid	Fibrous pseudocapsule	Desmin, actin, calponin positive, CD117, CD34 DOG-1 negative ki67/mib1 < 1%

With regard to macroscopic appearance, colon leiomyoma can be sessile (i.e., pedunculated, intraluminal, intramural, and extraluminal). The clinical picture is essentially non-specific, except in the case of large tumors, presenting occasional bleeding, a palpable and often prolapsing mass, and occasional pelvic or anal pain [9, 14].

The role of endoanal ultrasound is of utmost importance in characterizing the lesion since it is a simple, non-invasive, and cheap tool. Computed tomography and magnetic resonance imaging may be helpful in the diagnosis [10], but a high index of suspicion is necessary for a preoperative correct diagnosis. However, even if CT and MRI can give detailed features of the neoplasm, these information does not add any specific help in guiding surgical approach. Conversely, in expert hand, EAU is very effective in describing the relationships with the surrounding structure (anal sphincter, rectum) in addition to clinical signs (mobility, surface, shape). This is very helpful in deciding a straightforward surgical approach in order to relieve patients’ symptoms and obtain a correct diagnosis at pathology. Functional studies of the anal sphincter have not been performed and this may be a limitation of the clinical case. However, the patient did not report any continence impairment.

Histopathological classification (WHO classification of soft tissue tumors 5th Edition 2020) includes three types of angioleiomyoma: (1) solid type, characterized by closely compacted bundles of smooth muscle cells with few intervening, thin-walled, slit-like vascular channels; (2) venous type, with numerous thick walled blood vessels blending with smooth intervascular bundles; (3) cavernous type, composed of dilated vascular channels with a proliferation of smooth muscle bundles in the intervascular spaces. Tumors showing mixed morphologies may be seen [9, 26, 27]. Our patient had a venous-type angioleiomyoma.

According to Matsuyama and colleagues, expression of desmin in vascular smooth muscle cells also varies according to the anatomical site, the layer in the vascular wall, the kind or the size of the blood vessel, and the cellular condition such as in contractile or synthetic states [28].

## Conclusion

In conclusion, angioleiomyoma is a very rare neoplasm of the gastrointestinal tract and the fundamental problem of peri-rectal/perianal angioleiomyoma is represented by differential diagnosis from GISTs and other perianal/perirectal swellings. When such entity is encountered, a correct differential diagnosis at histopathology is supported by extensive immune-histochemical study adopting a panel of specific tissue markers. Considering the nature of such tumor, surgical treatment is mandatory with complete excision and subsequent follow-up since local recurrence may occur, although very rarely.

## Supplementary Information

Below is the link to the electronic supplementary material.

Supplementary Material 1. Care checklist has been used for the preparation of this case report CARE Case Report Guidelines (supplementary file). (PDF 644 KB)

## Data Availability

No datasets were generated or analysed during the current study.
